# Occurrence and Dissipation of the Antibiotics Sulfamethoxazole, Sulfadiazine, Trimethoprim, and Enrofloxacin in the Mekong Delta, Vietnam

**DOI:** 10.1371/journal.pone.0131855

**Published:** 2015-07-02

**Authors:** Chau Nguyen Dang Giang, Zita Sebesvari, Fabrice Renaud, Ingrid Rosendahl, Quang Hoang Minh, Wulf Amelung

**Affiliations:** 1 United Nations University, Institute for Environment and Human Security (UNU-EHS), Platz der Vereinten Nationen 1, 53113, Bonn, Germany; 2 Institute of Crop Science and Resource Conservation (INRES), Soil Science and Soil Ecology, University of Bonn, Nussallee 13, 53115, Bonn, Germany; 3 Hue University, College of Sciences, Department of Chemistry, Nguyen Hue 77, Hue, Vietnam; 4 Department of Natural Resources and Environment—Thua Thien Hue province, Center for Natural resource and Environment monitoring, Pham Van Dong 173, Hue, Vietnam; National Institute of Technology Rourkela, INDIA

## Abstract

The Mekong Delta in Vietnam has seen a rapid development and intensification of aquaculture in the last decades, with a corresponding widespread use of antibiotics. This study provides information on current antibiotic use in freshwater aquaculture, as well as on resulting antibiotic concentrations in the aquatic environment of the Mekong Delta. Two major production steps, fish hatcheries and mature fish cultivation, were surveyed (50 fish farm interviews) for antibiotic use. Different water sources, including surface water, groundwater and piped water (164 water samples) were systematically screened for antibiotic residues. To better understand antibiotic fate under tropical conditions, the dissipation behavior of selected antibiotics in the aquatic environment was investigated for the first time in mesocosm experiments. None of the investigated antibiotics were detected in groundwater and piped water samples. Surface water, which is still often used for drinking and domestic purposes by local populations, contained median concentrations of 21 ng L^-1^ sulfamethoxazole (SMX), 4 ng L^-1^ sulfadiazine (SDZ), 17 ng L^-1^ trimethoprim (TRIM), and 12 ng L^-1^ enrofloxacin (ENRO). These concentrations were lower than the predicted no effect concentrations (PNECs) and minimum inhibitory concentrations (MICs), suggesting limited antibiotic-related risk to aquatic ecosystems in the monitored systems. The dissipation half-lives of the studied antibiotics ranged from <1 to 44 days, depending on the availability of sunlight and sediment. Among the studied antibiotics TRIM was the most persistent in water systems. TRIM was not susceptible to photodegradation, while the dissipation of ENRO and SDZ was influenced by photolysis. The recorded dissipation models gave good predictions of the occurrence and concentrations of TRIM, ENRO and SDZ in surface water. In summary, the currently measured concentrations of the investigated antibiotics are unlikely to cause immediate risks to the aquatic environment, yet the persistence of these antibiotics is of concern and might lead to chronic exposure of aquatic organisms as well as humans.

## Introduction

Vietnam, followed by Indonesia, Bangladesh, Thailand, Myanmar and the Philippines, was the number one producer of aquaculture products in Southeast Asia in 2010. With an export volume of US$5.1 billion in 2010, Vietnam became the fourth-largest exporter in the world [1]. The total area used for marine and freshwater aquaculture in Vietnam was ca. 1.04 million ha in 2012, delivering more than 5.7 million tons of aquaculture products for both domestic food demand and export. The lower Mekong Delta (Vietnam) contributed 57% of this output in 2011 [2]. However, the economic benefit of aquaculture production is affected by disease outbreaks caused by bacterial infection, viruses or parasites. Therefore, chemical and biological products, particularly antibiotics, have been applied to aquaculture ponds to prevent and treat diseases and to promote growth. It was estimated that about 0.15 kg of medicine containing antibiotics was used for each ton of fish produced [3]. At the same time, fish farms were reported to discharge wastewater directly into the rivers (63%), and primary canals (19%) [4]. In the absence of treatment facilities, antibiotics are likely to reach the water system, causing water pollution and probably contributing to the development of resistant strains of bacteria (e.g., [5,6])

Antibiotic pollution has been assessed in few studies in the Mekong Delta. In mangrove areas located in Thai Binh, Nam Dinh, and Ca Mau provinces and in Can Gio forests, the highest residue concentrations found in mud samples were 0.73 g kg^-1^ for the antibiotic trimethoprim, 0.82 g kg^-1^ for sulfamethoxazole, 2.62 g kg^-1^ for norfloxacin and 0.43 g kg^-1^ for oxolinic acid. Water samples collected from shrimp ponds contained residues at maximum concentrations of 1.04 mg L^-1^ trimethoprim, 2.39 mg L^-1^ sulfamethoxazole, 6.06 mg L^-1^ norfloxacin and 2.50 mg L^-1^ oxolinic acid [7]. In 2007, sulfamethazine, sulfamethoxazole, trimethoprim and erythromycin-H_2_O were reported to occur at concentrations from 7 to 360 ng L^-1^ in surface waters collected in the Mekong Delta [8]. The same study also documented the concentration range of sulfamethazine in pig farm wastewaters and in canals near chicken and pig farms with concentrations of up to 19.2 10^3^ ngL^-1^. Knowing that inhabitants in rural areas frequently use surface water for domestic purposes and in some cases also for drinking (e.g., [9,10,11,12]), long-term chronic exposure to these antibiotics is likely to occur. So far, there are no guidelines for threshold concentrations of antibiotics in waters or soils.

After application, antibiotics can be transported through different pathways. Sorption has been considered as the major process governing the mobility and transport of antibiotics in environment [5], while in the meantime evidence is increasing that sequestration in particular, and bound-residue formation largely control the long-term storage of antibiotics in soils and sediments (e.g., [13,14,15]). The dissipation half-life of antibiotics may thus vary considerably, depending on the compound, degradation pathway and environmental media. Fluoroquinolones, for instance, maybe readily degraded by fungal cultures (e.g., [16,17]) or through photodegradation in aqueous media [18] but persist by strong sorption in soils for months [14]. Many of the antibiotic dissipation data stem from aerobic environments or laboratory studies that may not or only partially be translated to natural field conditions of aquaculture production systems (e.g., [19,20,21]) with varying water and air temperature, dilution by rainfall, presence of other microorganisms, and limited oxygen supply. Besides, most of those studies were conducted in temperate climate regions. The fate of oxytetracycline and oxolinic acid in an inland fish pond under tropical conditions was preliminary simulated by a modeling approach [22]. However, almost nothing is known about the dissipation at a natural field scale of antibiotics in a tropical climate, such as that prevailing in the aquaculture systems of large tropical deltas like in Vietnam.

Currently, information on antibiotic presence and concentration in the aquatic environment, particularly in drinking water sources, and the corresponding transport and dissipation characteristics have not been systematically studied, at least for tropical systems. Therefore, this research aimed to provide for the first time 1) a comprehensive overview of the use and environmental concentrations of common antibiotics being used in the two major production steps, fish hatcheries and mature fish, in the Mekong Delta, 2) assessment of the potential risk of antibiotic pollution to ecosystem and human health via different drinking water sources, and 3) empirical evidence of the fate of the selected target antibiotics sulfadiazine, trimethoprim and enrofloxacin under semi-field experimental conditions in a tropical climate.

## Material and Methods

### Study sites and sampling locations

**Fig 1:**
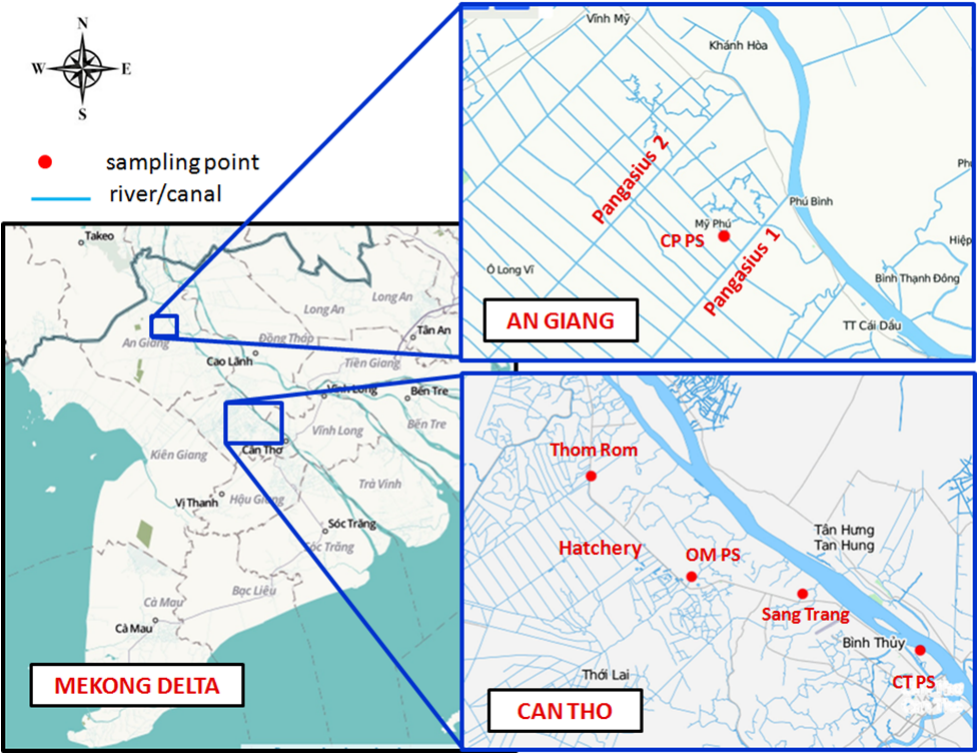
Study sites in Can Tho City and An Giang Province. *Map resource*: http://www.openstreetmap.org/.

The study was carried out in the Mekong Delta of Vietnam, specifically Can Tho City (in the middle of the delta) and An Giang Province (in the northern part of the delta) (Fig 1). The sampling sites were selected in order to cover different ranges of antibiotic use and potential environmental concentrations related to two major production steps in aquaculture: fish hatcheries and mature fish cultivation.

The first selected site was located close to the Hau (Bassac) River (one of the distributaries of the Mekong River) in Chau Phu district, An Giang Province. This site represented intensive cultivation of mature catfish. The total area of the study site was 140 hectares. During the time of this study (2011/2012), the area had 35 fish farms with an average farm size of 1.8 ha. Mature catfish were normally harvested after 8 to 10 months of cultivation, starting in March and harvested from October to November. Water was exchanged using electric pumps. The main domestic water sources in the area were piped water (30% interviewed households) and surface water (67%). Up to 33% of the respondents also reported using surface water for drinking and cooking (household survey result). Six sampling locations were identified in two parallel secondary canals (see Fig 1) named *Pangasius 1* (P1a: 10° 36' 50.00" N, 105° 12' 16.30" E; P1b: 10° 36' 23.48" N, 105° 11' 56.34" E; P1c: 10° 36' 00.93" N, 105° 11' 36.91" E) where aquaculture wastewater was discharged to the rice fields before reaching the canal and *Pangasius 2* (P2a: 10° 39' 03.41" N, 105° 10' 58.38" E; P2b: 10° 38' 36.54" N, 105° 10' 30.29" E; P2c: 10° 38' 08.51" N, 105° 10' 02.98" E), which received direct discharges from the catfish farms and also served as a domestic water source for local people.

The second site was located in Co Do, a rural district of Can Tho City. The site covered an area of 80 hectares with 10 fish farms with an average farm size of ca. 2.9 ha (including surface water area and dikes). The dominant aquaculture production step at this site was fish hatchery–mainly for carp, tench, tilapia, and catfish. The hatchery cycle lasted 2 to 3 months without a break between cycles. Nine of the ten farms discharged the wastewater directly into the adjacent canals. One farm had a lake to pre-treat wastewater before discharge. Paddy rice, upland crops and/or fruit trees surrounded the hatchery ponds. Groundwater and surface water were the main water sources for the inhabitants (70% and 40% of respondents, respectively) (household survey of this study, see also [23]). Three sampling locations were set in a secondary canal (see Fig 1) named *Hatchery* (H1: 10° 08' 21.21" N, 105° 33' 43.22" E, H2: 10° 08' 2.17" N, 105° 33' 55.84" E, and H3: 10° 07' 36.98" N, 105° 34' 11.54" E) where local people extracted water for domestic purposes.

Additionally, three sampling locations were set at the intake points of public pumping stations (PSs), which sourced water for a piped water supply station, located in 1) Hau river (CTPS: 10° 04' 14.99" N, 105° 46' 12.01" E) in Can Tho City, 2) O Mon river (OMPS: 10° 06' 54.96" N, 105° 37' 02.46" E) in O Mon district, (also Can Tho City), and 3) a secondary canal in Chau Phu district (An Giang Province) (CPPS, 10° 36' 16.85" N, 105° 11' 15.42" E). Moreover, two additional locations were selected in the two main canals in Can Tho City, i.e. the Sang Trang canal (ST: 10° 06' 07.97" N, 105° 41' 31.59" E) and the Thom Rom canal (TR: 10° 10' 58.67" N, 105° 33' 01.66" E), where the Sang Trang canal received industrial and domestic effluents while the Thom Rom canal received aquaculture wastewater and domestic effluents (expert interview).

### Selected antibiotics and monitoring campaigns

#### Household interview

Questionnaire based household surveys were conducted in both study areas with the approval of the Department of Natural Resources and Environment (DONRE) of Can Tho City and the DONRE of An Giang Province. A semi-structured questionnaire was applied, which consisted of two main sections. The first section focused on the general demographics of the households, the water sources used for drinking, and general water consumption patterns. The second section focused on antibiotic use and fish farm characteristics. Twenty interviews were carried out at the *Hatchery* site (Can Tho)—in September 2011, and 30 at the *Pangasius* site (An Giang)—in March 2012. Respondents provided their written informed consent to participate in this study. Adequate information for the second section was, however, only received from 7 households at the *Hatchery* site and 10 households at the *Pangasius* sites. The fish pond owners hesitated in some cases to answer questions related to antibiotic use and farm operation.

#### Selection of studied antibiotics

Based on initial field survey results, the most common antibiotics used in aquaculture in these two study provinces were enrofloxacin (belonging to the fluoroquinolones, used in 47% of the farms), trimethoprim (diaminopyrimidine, 41%), sulfamethoxazole (sulfonamides, 41%), doxycycline (tetracyclines, 29%), florfenicol (phenicol, 29%), sulfadiazine (sulfonamides, 18%), amoxicillin (penicillins, 18%), ampicillin (penicillins, 12%), and oxytetracycline (tetracyclines, 12%). A minority of farmers reported using spectinomycine (aminoglycosides, 5.9%), sulfadimethoxine (sulfonamides, 5.9%), cephalexin monohydrate (penicillins, 5.9%), kanamycin sulfate (aminoglycosides, 5.9%), and chloramphenicol (phenicol, 5.9%). Even though tetracyclines were used frequently, their sampling and analysis requires avoiding glassware and using plastic apparatus due to their strong binding to borosilicate glassware [24], which prevented the monitoring and simultaneous determination of other antibiotics. Therefore, after consideration of usage frequency, physico-chemical properties (water solubility, octanol-water partition coefficient, soil organic carbon-water partition coefficient, acid dissociation constant, Table 1), and access to analytical equipment, sulfamethoxazole (SMX), sulfadiazine (SDZ), trimethoprim (TRIM) and enrofloxacine (ENRO) were selected for monitoring in water. For the fate study the same antibiotics except for SMX were considered. SMX has a similar mode of action as SDZ but it is less likely to cause harm to aquatic life (much higher median effective concentration (EC50) and median lethal concentration (LC50) concentrations compared to the other antibiotics, Table 2).

**Table 1:** Physicochemical properties of the studied antibiotics.

Antibiotics	Class	Water solubility	LogK_ow_	K_oc_	pKa	Use^d^
	* *	20^°^C (mg L^-1^)		(L kg^-1^)		(%)
Sulfamethoxazole^a^	Sulfonamides	2800	0.89	219	5.8; 1.4	41
Sulfadiazine	Sulfonamides	77^b^	-0.09^b^	124^c^	6.36^b^	17
Trimethoprim^a^	Diaminopyrimidines	1000	0.59	301	7.0	41
Enrofloxacine^a^	Fluoroquinolones	100	2.32	2179	6.4; 7.8	47

^*a*^ *: [25];*

^*b*^ *: [26];*

^*c*^ *: [5]*

^*d*^: *field survey*, *2011–2012*

K_ow_: octanol-water partition coefficient; K_oc_: soil organic carbon—water partition coefficient; pKa: acid dissociation constant

**Table 2:** Toxicity data for different aquatic organisms exposed to the studied antibiotics, PNEC, PEC, ratio of maximum quantification concentration and PNEC, and ratio of PEC and PNEC.

	Species	Test	Duration	Conc.	Sources	AF	PNEC	Max. quant. conc.	Max. quant. conc./PNEC	PEC		PEC/PNEC	
			(h)	(mg L^-1^)			(µg L^-1^)	(ng L^-1^)		(µg L^-1^)	(µg L^-1^)		
										*Pangasius*	*Hatchery*	*Pangasius*	*Hatchery*
**SMX**	Daphnia	EC50	96	177–204	[27]								
	Danio rerio (fish)	LC50	96	>1000	[27]	1000	562.5	239	0.0004	64	21	0.11	0.04
	Oryzias latipes (fish)	LC50	96	562.5	[27]								
**SDZ**	Daphnia magna	EC50	48	>57	[28]	100	10	108	0.0108	64	21	6.41	2.07
	Algae	NOEC	96	<1	[28]								
**TRIM**	Daphnia magna	LC50	48	123	[28]								
	Oryzias latipes(fish)	LC50	96	>100	[28]	100	255	330	0.0013	16	5	0.06	0.02
	Algae	NOEC	96	25.5	[28]								
**ENRO**	Oryzias latipes (fish)	LC50	96	10	[28]								
	Oryzias latipes (fish)	NOEC	21 days	1	[28]	50	20	81	0.0040	36	11	1.78	0.57
	Daphnia magna	NOEC	21 days	1	[28]								

EC50: median effective concentration; LC50: median lethal concentration; NOEC: no observed effect concentration; PNEC: predicted no effect concentration; PEC: predicted environmental concentration; AF: assessment factor

#### Antibiotic monitoring strategy

A total of 154 surface water samples were collected at 14 selected locations in eleven sampling events performed monthly from March 2012 to January 2013 with the permission of the DONRE of Can Tho City and the DONRE of An Giang Province. Focusing on drinking water quality, sampling locations and times were set to represent the surface water extraction routine of local households, e.g. at 3 m distance from the canal bank during high tide (when residents expect to extract water of better quality). Water samples taken to represent the water utilized by the water supply stations were collected close to the inlet of the pumping stations.

Field survey results showed that a larger proportion of piped water was consumed at the *Pangasius* sites (in An Giang) whereas a larger proportion of groundwater was consumed at the *Hatchery* site (in Can Tho). To initially screen piped water quality at the *Pangasius* sites and groundwater quality at the hatchery site, 5 piped water samples were collected from household taps and 5 groundwater samples from private drill wells. The water was purged for 5 minutes before sampling.

Samples (500 mL) were collected in pre-cleaned glass bottles, acidified (pH 2.5), transported to the laboratory under cool conditions and extracted within 24 hours. For each sampling event, basic water quality parameters including pH, dissolved oxygen (DO), electrical conductivity (EC), and temperature were measured in-situ by a WTW Multi 340i instrument (Weilheim, Germany) at every sampling location.

### Experimental design of the fate study

The fate study was conducted under semi-field conditions. A secondary canal (10° 3' 17.27" N, 105° 42' 50.28" E) located in Binh Thuy district, Can Tho City was selected for the fate study. The canal was representative of the environmental conditions of the region, i.e. the water of the canal had a typically high electrical conductivity (EC) of 170 to 220 μS cm^-1^, while pH ranged from 6.8 to 7.2, and a low dissolved oxygen concentration (2 to 4 mg L^-1^) prevailed. Additionally, this site was surrounded by an area where no significant antibiotic pollution was expected from the region, i.e. the main land-use in this area was rice cultivation and fruit production, with no aquaculture or animal husbandry within a radius of 1 km. Moreover, it allowed for fast sample transportation to the laboratory (< 10 km distance). Finally, the site was located in an area where local authorities and farmers were willing to cooperate in setting up the experiment and to protect the microcosms against physical damage during the study period. The canal received water from a primary canal which was directly connected to the Hau River. The canal was influenced by tides. The difference between the lowest and highest water level during the experimental period was about 1.5 m.

**Fig 2:**
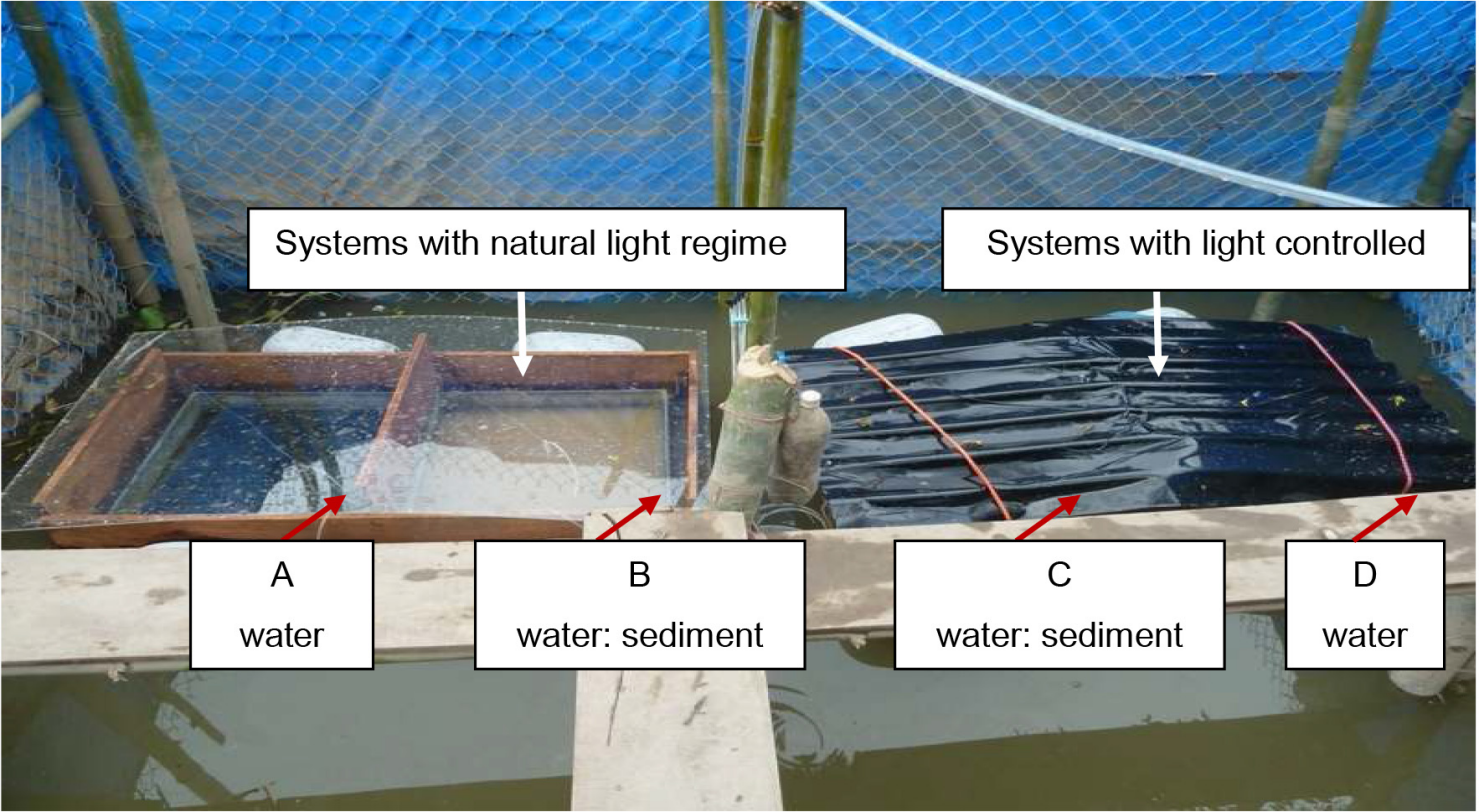
Experiment set up of the microcosms for assessing the dissipation of antibiotics in water and water:sediment systems.

The semi-field experiment was designed based on a study by Laabs et al. [29] with 4 different microcosm systems (Fig 2):

- water system with natural light regime (system A),
- water:sediment system with natural light regime (system B),
- water:sediment with light control (system C),
- water system with light control (system D)

Microcosms (60 x 60 x 54 cm) were constructed from glass plates of 5 mm thickness. Black glass plates were used for systems with light control. The systems were filled with filtered canal water (filtration by a stone layer and a sand layer) from the adjacent primary canal. The total water volume in the water systems was 194.4 L. The sediment to water ratio in sediment:water systems was 1:5 (v:v), corresponding to 9 cm sediment and 45 cm water layer (ca. 162 L of water). The sediment to set up the systems originated from an adjacent primary canal taken from a depth of 20–30 cm. The sediment was homogenized prior to use, and stones, leaves, and other materials were removed. The system was allowed 3 days to reach equilibrium, after which the basic characteristics of the water and sediment were analyzed (see S1 Table). Antibiotic solutions for initial spiking of the systems were prepared in a 50 mL methanol:water 50:50 (v:v) mixture, in which the initial concentration was ca. 20 10^3^ ng L^-1^ for each antibiotic (20.09 10^3^ ng L^-1^ of SDZ, 19.59 10^3^ ng L^-1^ of ENRO, and 21.71 10^3^ ng L^-1^ of TRIM). Accordingly, the volume of methanol introduced to each system after spiking was 6.25 mL (accounting for 0.00315%, which should not influence the dissipation of studied antibiotics in water). The water phase was stirred during the application of antibiotics but the sediment layer was not disturbed.

Wooden frames connected with plastic cans were constructed to keep the systems floating at the same water level as the canal water. Bamboo pillars and iron steel B40 nets were used as a fence to protect the systems against debris or water waves caused by boats. The concentration of dissolved oxygen, pH, electrical conductivity (EC) and water temperature were measured at 10 cm and 45 cm water depths in the test systems at every sampling date by a WTW Multi 340i instrument (Weilheim, Germany).Water loss due to evaporation during the fate study was compensated for with distilled water.

Samples were collected from each system in ten sampling events in three replicates. Sampling dates were: 0, 1, 2, 4, 8, 15, 29, 43, 57 and 85 days after application, starting on 14 May2012 and completed on 7 August 2012.

### Antibiotic analysis

#### Chemicals and reagents

Antibiotic standards of sulfadiazine, sulfamethoxazole, trimethorprim and enrofloxacin were obtained from Sigma-Aldrich (Seelze and Schnelldorf, Germany). The internal standards including isotope-labeled ^13^C_6_-sulfadiazine were provided by the Institute of Environmental Biology and Chemodynamics of the RWTH Aachen University, while enrofloxacin hydrochloride (ethyl-d5), ^13^C_6_-sulfamethoxazole and ^13^C_6_-trimethoprim were supplied by LGC Standards (Wesel, Germany). All employed solvents were of HPLC grade. For accelerated solvent extraction (ASE), sea sand, salts, and acids were pro-analysis grade. A Millipore Synergy water treatment system was used to produce water.

#### Analytical method

Antibiotics in the water samples were analyzed according to modified methods developed by Gobel et al. [30]. Sample preparation was conducted in the Advanced Laboratory, Can Tho University, Vietnam. Solid-phase extraction was employed using Chromabond SB (SAX, Macherey & Nagel, Germany) in combination with Oasis HLB (Waters, USA) to reduce matrix effects and clogging. The cartridges were preconditioned with 4 mL methanol and 4 mL Millipore water at pH 2.5. A 500 mL water sample was filtered through a glass fiber filter (pore size 1 μm) before being applied to the cartridge at a flow rate of ca. 1.5 mL min^-1^. The cartridge was then washed with 5 mL Millipore water and dried in a nitrogen flow for 30 minutes. Dry cartridges were kept frozen at -40°C, which then were transferred frozen to the laboratory of the Division of Soil Science and Soil Ecology, Faculty of Agriculture, University of Bonn, Germany for elution and measurement. The analytes adsorbed on the solid phase of the Oasis HLB cartridge were eluted sequentially by 4 mL methanol, 4 mL acetonitrile and 4 mL acetonitrile + 0.1% HCl. The eluate was concentrated to ca. 500 μL by rotary evaporation and transferred to amber vials containing 25 ng of the internal standards ^13^C-SMX, ^13^C-SDZ, ^13^C-TRIM and ENRO-D5 hydrochloride. Amber vials were filled up to ca. 1 mL with 50 mM phosphoric acid:acetonitrile (80:20) solution and stored at -20°C until measurement.

The sediment samples from the dissipation study were stored at -20°C in the Advance Laboratory, Can Tho University, Vietnam and transferred frozen to the laboratory of the Division of Soil Science and Soil Ecology, Faculty of Agriculture, University of Bonn, Germany. Sediments were lyophilized and sieved to a grain size < 2 mm. Ten g of dry material of each sediment sample mixed with sea sand was extracted employing accelerated solvent extraction (ASE). Two different solutions for the extraction were combined to account for the different physico-chemical properties of the antibiotics (Table 1). Anaqueous 50 mM phosphoric acid:acetonitrile solution (50:50, v:v) adopted from Golet et al. [31] and a methanol:water solution (50:50, v:v) from Gobel et al. [30] were used. Twenty mL of ASE extract was diluted to 400 mL by Millipore water and adjusted to pH 2.5. The analytes were then extracted and eluted using the same procedure described above for the water samples.

Antibiotic concentrations were analysed by liquid chromatography coupled with tandem mass spectrometry (LC-MS/MS, Quantum Ultra; Thermo Electron Corporation, Dreieich, Germany), equipped with a heated electro spray ionisation source (ESI) in the positive ion mode with an injection volume of 10 μL. Antibiotic classes were separated by XBridge C18 collumn (3.5 μm, 2.1x150 mm, Waters, USA) with the following mobile phases: solvent A: acetonitrile + 0.1% HCOOH, solvent B: 1 mM ammonium acetate in millipore water + 0.1% HCOOH with 400 μL min^-1^ flow. The initial condition was 5% A and 95% B, changed to 60% A over 5 min, then to 80% A over 10 min, then to 100% A over 1 min, hold 100% A for 2 min and return to the initial condition, hold initial condition for 7 min. The current was set at 4000 V, the vaporizer temperature was 390°C and the capillary temperature was 217°C.

Routine limit of quantification (RLOQ = lowest concentration of standard used) was calculated at 1 ng L^-1^ water and 50 ng kg^-1^ sediment dry weight for all studied antibiotics.

The recovery rates of antibiotics from water samples (n = 5) were 63 (± 6.6) % for ENRO, 77.6 (± 4.5) % for SDZ, SMX and TRIM. In the dissipation study, the recovery rates for spiked sediment samples (n = 3) ranged from 32% to 42% for ENRO, and from 76% to 93% for SDZ and TRIM. Monitored concentrations were not corrected for recovery rates.

### Statistical methods

IBM SPSS Statistics version 20.0 (IBM Corp, Armonk, New York, USA) and Sigma Plot version 11.0 softwares (Systat Software Inc, San Jose, California, USA) were used to perform the statistical analysis. The Kolmogorov–Smirnov test was applied to test the normal distribution of the data (p = 0.05). If the data was normally distributed, one way ANOVA was run to find significant differences between sites. Otherwise non-parametric tests were used. The Kruskal-Wallis H test was applied to find differences between sites**.** The Mann–Whitney U tests were used to identify differences within sites.

Statistics for fate study

Exponential decay equations were fitted to antibiotics dissipation data:

$${\text{C}}_{\text{t}}={\text{C}}_{0}{\text{e}}^{\left(-\text{kt}\right)}$$

C_t_: the concentration of antibiotic at time t (ng L^-1^ or ng kg^-1^)

C_0_: the initial concentration (ng L^-1^)

k: the dissipation rate constant (d^-1^)

t: the elapsed time (d)

Dissipation half-lives (DT_50_: the time required for 50% of the initial concentration to dissipate) were calculated from the above equation: DT_50_ = ln(2) k^-1^. For persistence comparisons, 90% and 99% dissipation times were also calculated.

## Results and Discussion

### Fish farming practices and antibiotic use at the study sites

The interviews revealed that farmers at the *Hatchery* site (Can Tho) cultured ca. 2.6 million fingerlings per hectare with an average production yield from each cycle of ca. 40 tons ha^-1^ (ca. 30 g weight at the time of harvest; Table 3). At the mature *Pangasius* sites (An Giang), average fish density at the stocking stage was lower than at the *Hatchery* site (Table 3). At the time of harvesting, production yield was ca. 250 tons ha^-1^ per season (from 800 to 1000 g weight at the time of harvest). Mortality at the hatcheries was typically high (40%–50% per crop) due to the susceptibility of fingerlings to diseases. Overall, around 30% of the mature catfish was lost. This loss predominantly took place at the early stages of a cycle (own survey, and [4]). Prevalent fish bacterial diseases recorded at both sites were 1) red spot disease, with the occurrence of hemorrhages on head, mouth and fins, 2) necrosis, causing spots on liver, kidney, spleen. These were considered the most common and severe diseases of catfish in Vietnam (e.g., [32,4]). Fish farmers at both sites assumed that mainly specific weather conditions, e.g. the floods and heavy rain which take place annually from August to November were responsible for the spread of diseases. Local farmers buried or even sold the dead fish to local dealers, which likely contributed to the spread of the diseases [4]. High loss rates caused by diseases in combination with high (but also fluctuating) market demand were thus mentioned by farmers as reasons for antibiotic use. Additionally, antibiotics were also applied prophylactically (own survey, and [33]).

**Table 3:** Fish farming and antibiotic use in the *Hatchery* and *Pangasius* sites.

	*Hatchery* site	*Pangasius* sites
	(n = 7)	(n = 10)
^(^ ^*^ ^)^Ave. farm size (ha)	2.9	1.8
Ave. fish weight at harvest time (g)	30	960
Ave. initial stock density (individuals ha^-1^)	2,700,000	374,000
Ave. production (tons ha^-1^) per crop	40	250
Loss rate per crop (%)	40–50	30
Water exchange frequency (days)	35	Everyday, partial exchange
Pond depth (m)	1–1.5	2.5–5
Antibiotic use technique	Instruction on label, Antibiotic retailers	Aquaculture extensionists
Farmer/worker directly exposed to antibiotics (dermal exposure)	100%	100%
Av. number of different types of antibiotics used per season	2	3
Overdose application (based on farmer´s report)	40%	Farmers did not answer

^(*)^ Each fish farm could have one or more fish ponds

Among the 14 currently used antibiotics at the two study sites, ENRO, SMX, TRIM, doxycycline and florfenicol were most often used (see also Selection of studied antibiotics). Notably, ENRO and chloramphenicol are prohibited in aquaculture production according to Vietnam's Circular 15/2009/TT-BNN promulgated by the Ministry of Agriculture and Rural development [34]. Farmers at both sites used their unprotected hands to mix antibiotics with food or water (in case of bath treatments). In general, two different antibiotic active ingredients were applied per hatchery farm and three at the mature *Pangasius* farm. At the *Hatchery* site, 40% of farmers reported applying a higher dosage than the recommendation on the product's label. No information could be collected on the antibiotic dosage applied at the mature *Pangasius* sites because local fish farmers refused to answer and asserted that they had completely followed the instructions of aquaculture extension officers. The same situation was also reported by Tuan and Munekage [7] who faced difficulties when attempting to collect information on the dosage of antibiotics used in the case of shrimp farmers. Despite the information gaps, the interview results demonstrated a tendency of inadequate or at least uninformed antibiotic handling.

At the *Hatchery* farms, the water depths in ponds were low and varied from 1 to 1.5 m. The farms discharged water from the ponds every 35 days on average. Water was taken in or discharged out from the same canal (where sampling locations were set). Water treatment before discharge was only reported at one farm, in which wastewater was drained to a lake for sedimentation prior to lime application. This low rate of water treatment was also reported in the study of Lam et al. [4], in which only 11.2% of investigated catfish farms applied chlorine or lime before discharging wastewater. At the mature *Pangasius* farms, pond depth ranged from 2.5 to 5 m. Ponds were surrounded by dikes, which were built to prevent the loss of fish during the flooding season or heavy rainfall events (household interview, and [35]). Pond water was partly refreshed daily. Similar practices were also mentioned by Carballo et al. [36] and Lam et al. [4]. The wastewater from these mature fish farms was normally discharged to the surrounding fields or in most of the cases at the site *Pangasius 2* to the adjacent canals.

In general, these farming practices in combination with untreated discharge flows from fish ponds containing excess feed, dead fish and residues of veterinary medicines including antibiotics suggested a risk of pollution of the surrounding aquatic environments.

### Occurrence of antibiotics in surface water

As shown in Table 4, almost all surface water samples were contaminated by at least one antibiotic (91.6%), while in 55.2% of the samples, a mixture of 3 or 4 antibiotics co-occurred. Among the two studied sulfonamides, SMX was more frequently detected in surface water than SDZ (82% vs. 58%, respectively). Detected concentrations of SMX ranged from 1 to 239 ng L^-1^ (median 21 ng L^-1^). These concentrations were lower than the concentrations reported by Managaki et al. [8], where median concentrations of SMX were 80 ng L^-1^. The concentrations of SDZ were lower compared with that of the other antibiotics (Table 4). This is in line with interview results showing that SDZ was less frequently used than the other antibiotics (Table 1). The highest SDZ concentration of 108 ng L^-1^ was observed in the sample from the *Hatchery* site in June 2012. In terms of veterinary medicine for fish, SMZ and TRIM or SMX and TRIM are normally mixed in commercial products, e.g. in "Trimesul" and "Cotrim" (field survey) since the effectiveness of sulfonamides is enhanced when combined with TRIM [37]. Hence, TRIM was also abundant and occurred in 87% of monitored water samples (Table 4). This diaminopyrimidine compound was quantified with a median concentration of 17 ng L^-1^ which was comparable to the median concentration (20 ng L^-1^) reported by Managaki et al. [8], suggesting that the concentrations found here are in a typical range. SMX and TRIM residues in water taken from shrimp farms in the South of Vietnam [7] were one order of magnitude higher. In Taiwan, concentrations of SMX and TRIM in aquaculture wastewater were higher (median 229 ng L^-1^ and 85 ng L^-1^, respectively) [38]. ENRO is forbidden and thus has the lowest detection frequency. Nevertheless, 38% of surface water samples contained this compound with median concentration of 12 ng L^-1^ (Table 4). Low water solubility in combination with strong adsorption to soil of the fluoroquinolone group in general (e.g., [31,14]) and of ENRO in particular (Table 1) were the main factors limiting the detection of ENRO in water.

**Table 4:** Occurrence of SMX, TRIM, SDZ, ENRO ng L^-1^ in collected surface water samples in 2012.

		*Hatchery*			*Pangasius 1*			*Pangasius* 2			Pumping stations (PSs)			Main canals		TOTAL
	Sampling locations ^(^ ^*^ ^)^	H1	H2	H3	P1a	P1b	P1c	P2a	P2b	P2c	CP PS	OM PS	CT PS	Sang Trang	Thom Rom	
**ENRO**	Mar	7	-	-	-	-	-	-	-	-	5	-	-	-	6	
(n = 154)	Apr	-	12	4	-	1	4	6	4	7	8	10	6	6	5	
	May	-	-	17	-	-	5	-	-	6	5	-	5	-	10	
	Jun	26	14	12	22	28	16	59	18	25	34	28	40	22	55	
	Jul	34	30	-	15	11	17	20	43	20	15	9	20	12	-	
	Aug	-	-	-	-	-	-	-	-	-	-	-	-	-	-	
	Sep	-	-	-	-	-	-	-	-	-	-	-	5	5	-	
	Oct	-	-	-	24	-	-	-	19	5	49	17	-	-	15	
	Nov	-	-	-	5	-	11	-	-	-	-	-	-	8	-	
	Dec	-	-	-	-	-	-	-	-	3	-	-	-	-	-	
	Jan ^(^ ^*^ ^)^	-	-	-	-	-	-	-	-	-	-	-	-	-	-	
	**Median**		**14**			**13**			**18**		**11**	**13**	**6**	**8**	**10**	**12**
	**Quant. freq (%)**		**27.3**			**36.4**			**39.4**		**54.5**	**36.4**	**45.5**	**45.5**	**45.5**	**38.3**
**SDZ**	Mar ^(^ ^***^ ^)^															
(n = 126)	Apr ^(^ ^***^ ^)^															
	May	3	4	6	-	2	2	4	8	4	2	4	11	14	2	
	Jun	108	22	90	2	-	3	6	10	10	6	48	17	36	26	
	Jul	-	4	-	-	-	-	-	-	-	-	-	-	-	-	
	Aug	6	6	13	2	-	-	25	9	2	4	1	-	21	3	
	Sep	14	4	7	-	-	-	2	2	2	-	3	4	12	3	
	Oct	2	2	2	17	-	1	4	10	4	15	11	2	3	10	
	Nov	1	-	-	3	2	7	3	-	3	1	3	3	63	8	
	Dec	-	-	-	-	-	-	-	-	-	-	-	-	-	-	
	Jan ^(^ ^*^ ^)^	-	-	-	-	-	-	-	-	-	-	-	-	12	-	
	**Median**		**6**			**3**			**5**		**4**	**4**	**4**	**14**	**6**	**4**
	**Quant. freq (%)**		**63.0**			**37.0**			**63.0**		**55.6**	**66.7**	**55.6**	**77.8**	**66.7**	**57.9**
**SMX**	Mar	10	2	16	15	27	10	40	94	-	22	22	80	148	6	
(n = 154)	Apr	-	10	-	81	-	3	6	6	16	-	4	6	-	-	
	May	46	104	42	20	27	32	48	44	92	40	25	80	143	3	
	Jun	30	21	24	14	34	38	74	88	88	76	21	34	55	39	
	Jul	19	34	15	17	12	11	13	8	25	18	6	8	5	156	
	Aug	38	29	58	20	17	14	24	17	4	21	16	-	239	43	
	Sep	44	11	32	9	5	33	8	12	10	36	9	20	3	14	
	Oct	47	80	135	10	6	10	92	73	75	10	70	51	57	185	
	Nov	21	-	20	3	7	28	22	19	14	10	13	17	19	31	
	Dec	-	-	-	9	2	3	11	9	45	-	3	-	34	-	
	Jan ^(^ ^*^ ^)^	-	-	-	3	-	8	-	-	-	-	-	-	-	-	
	**Median**		**31** ^**a**^			**12** ^**b**^			**22** ^**a**^		**21**	**15**	**27**	**55**	**35**	**21**
	**Quant. freq (%)**		**72.7**			**93.9**			**87.9**		**72.7**	**90.9**	**81.8**	**81.8**	**72.7**	**82.5**
**TRIM**	Mar ^(^ ^***^ ^)^															
(n = 126)	Apr ^(^ ^***^ ^)^															
	May	29	24	6	24	26	26	36	30	50	17	19	40	41	24	
	Jun	69	23	309	12	16	26	41	42	46	49	144	104	111	78	
	Jul	71	330	22	13	8	12	32	29	46	10	37	24	45	163	
	Aug	14	11	16	18	6	5	11	12	-	10	13	-	45	20	
	Sep	18	23	63	7	1	19	11	14	13	21	9	9	11	23	
	Oct	13	16	15	7	5	30	37	25	16	13	20	14	21	21	
	Nov	13	-	20	7	9	18	14	16	17	8	17	15	23	20	
	Dec	-	-	-	5	-	-	-	-	1	-	6	1	11	-	
	Jan^(^ ^*^ ^)^	-	-	-	14	13	7	9	13	6	3	2	1	3	-	
	**Median**		**0.021** ^**a**^			**0.012** ^**b**^			**17** ^**a**^		**12**	**17**	**14**	**23**	**23**	**17**
	**Quant. freq (%)**		**74.1**			**92.6**			**88.9**		**88.9**	**100.0**	**88.9**	**100.0**	**77.8**	**87.3**

^(*):^ Sampling locations: see section "Study sites and sampling locations" for details

^(**)^: January 2013

^(***):^ not monitored

-: no detection

^a^ and ^b^: median concentrations of individual antibiotics with different letter superscript indicate significant differences between study sites (p < 0.001)

#### Antibiotic residues in water at the fish farm sites

Spatial variation: Significantly lower median concentrations of SMX and TRIM were found (p < 0.001) at the site *Pangasius 1* (12 ng L^-1^ for both antibiotics) in comparison with the site *Pangasius 2* (median 22 ng L^-1^ for SMX and 17 ng L^-1^ for TRIM) and the *Hatchery* site (median 31 ng L^-1^ for SMX and 21 ng L^-1^ for TRIM). The results suggest that antibiotic contamination in water was partly mitigated by draining wastewater to the rice fields (see *Pangasius 1*) prior to discharge to surrounding aquatic environments. The drainage removed excess nutrients from aquaculture effluents effectively, while serving as a fertilizer in rice production (e.g., [39,40]).

SMX and TRIM occurred more often in water samples collected from *Pangasius* than from *Hatchery* sites (Table 4). This might partially be due to the more frequent effluent discharge at the *Pangasius* sites than at the *Hatchery* site (everyday vs. every 35 days, Table 3). Quantification frequency was similar for these two antibiotics at each site. This result is in line with the survey data showing that these two compounds have been applied in a mixture to enhance treatment efficiency.

As already reported for the surface waters, SDZ and ENRO residue levels were lower than that of the other antibiotics at both the *Hatchery* site and the *Pangasius* sites, ranging from 1 to 108 ng L^-1^ for SDZ and from 1 to 59 ng L^-1^ for ENRO, respectively (Table 4). The reasons are again related to lower application amounts (for SDZ) and the lower water solubility of both compounds (Table 1; see also discussion above).

Temporal variation: At both *Hatchery* and *Pangasius sites* ENRO was most frequently detected from April to July (79.4% of the contaminated water samples were sampled in these months). Almost 90% of the samples with ENRO concentrations higher than the median value (12 ng L^-1^), fell within this period. ENRO was almost completely absent in the samples taken between August 2012 and January 2013. The pattern for SDZ, SMX and TRIM was partly different. The median values (4 ng L^-1^ for SDZ, 21 ng L^-1^ for SMX, and 17 ng L^-1^ for TRIM) were exceeded in the water samples from May to November. An exception was the case of SDZ in July, in which the median value was not exceeded in any analyzed sample. Antibiotics were detected at low frequency and concentration in December 2012 and January 2013 (Table 4). This could be partly explained by the climate of the Mekong Delta. The period from May to October corresponds to the rainy season, with hot, humid and rainy weather (75% of total rain water fell in this season in 2012 (Ca Mau station, [41])). These climatic conditions generally facilitate diseases outbreaks and transmission, forcing fish farmers to use more antibiotics.

#### Antibiotic residues at the inlet of public pumping stations and in two main canals in Can Tho

At the investigated pumping stations, sampling points were set right at the inlet of the stations. The surface water extracted served as the source for piped water supply. For extracted water, treatments included flocculation, deposition, filtration, and chlorine disinfection in the water plant. These treatment methods are known to be able to remove several organic compounds [42].

During the eleven sampling campaigns from March 2012 to January 2013, SMX and TRIM were again detected at a higher frequency at all sampling points than SDZ and ENRO (Table 4). Except for the median concentration of SMX (21 ng L^-1^), which was significantly higher (p < 0.05) than that of SDZ (4 ng L^-1^) at CPPS, there were no statistically significant differences between occurrences of studied antibiotics within the inlet points of the monitored stations, or of specific antibiotics between stations. The prevalence of antibiotics at the CPPS (in a secondary canal in An Giang Province) and in the Thom Rom canal (a main canal in Can Tho City) could be caused by wastewater discharged from the surrounding intensive aquaculture farms. However, even though it was expected that less pollution would occur at the CTPS (located at the Hau River, one tributary of the Mekong River with high dilution capacity), antibiotics were also frequently detected (Table 4). The source of pollution at this sampling point could be attributed to the discharges from cage aquaculture cultivation along the upstream banks of the Hau River. The high solubility of antibiotics and the high water flows of the river would facilitate the transportation of antibiotics downstream to this pumping station. In the cases of the OMPS and Sang Trang canal, the field survey recorded a predominance of industrial and residential land-use. Therefore, the possible pollution sources could be from human pharmaceutical use or veterinary products from livestock farms. Since similar concentrations of antibiotics were found at all pumping stations, regardless of local differences in industry and land use, it seems likely that the captured concentrations of antibiotics in both incoming and discharging water sources are typical steady-state concentrations, and that the main sources of antibiotics in the region, including inland aquaculture, cage aquaculture, veterinary and human pharmaceutical products, have contributed to a region-wide occurrence of antibiotics that was detected in every waterway monitored.

Antibiotics were not detected in any of the collected ground- or piped water samples. However, the year-round prevalence of the studied antibiotics in surface water, together with the use of surface water for domestic and drinking water still gives rise to concerns as to whether—at least locally—antibiotic residues pose a risk for ecosystem and human health.

### Risk assessment

To assess the potential risk associated with antibiotic residues in the aquatic environment, the monitoring results were compared with Predicted No Effect Concentrations (PNEC) for aquatic organisms (e.g. [43]), and Minimum Inhibitory Concentration (MIC) for bacteria strains. Worst-case scenario was also conducted by calculating the Predicted Environmental Concentrations (PECs) of individual antibiotics based on the prescribed dosages from veterinary producers and water volumes in fish ponds and then compared to PNEC [44] and MIC values.

In terms of PNEC assessment, concentrations lower than the PNEC are assumed to pose no risk to the environment [45]. The toxicity data of individual studied antibiotics consisting of different EC (effect concentration) or LC (lethal concentration) values and NOECs (no effect concentrations) are shown in Table 2. To reflect the degree of uncertainty when extrapolating from available laboratory toxicity tests to effects in environment, so-called assessment factors (AFs) were used. Selection of these AF varied from 1000 to 1, based on European Commission guidelines ([45], see S2 Table for details). Where possible, the NOEC was used, since it is the more conservative value. PNEC value was calculated by dividing the lowest toxicity concentration of an antibiotic by the respective AF. In the case of SMX, since there was no NOEC value available, an AF of 1000 was selected. Meanwhile, only one NOEC for algae was found for SDZ and TRIM, thus the respective AF was 100. Of the four monitored antibiotics, ENRO was considered as the most toxic antibiotic to aquatic living organisms as there were at least two NOEC values available (see S2 Table). Therefore, the selected AF for ENRO was 50 (Table 2).

With regard to MIC data, there is a strong variation in the sensitivity of different bacterial strains to a certain antibiotic; hence, the reported MIC values of each studied antibiotic varied across abroad range (Table 5).

**Table 5:** Minimum inhibitory concentrations (MICs) for ENRO, SMX, TRIM and SDZ against some bacterial strains, ratio of max. quantification concentrations and MIC_50_, and ratio of PEC and MIC_50_.

		MIC			Source	Max. quant. conc.	Max. quant. conc./MIC_50_	PEC/MIC_50_	
		(mg L^-1^)				(ng L^-1^)		*Pangasius*	*Hatchery*
		E. coli	Gram possitive bacteria (231)	Gram neggative bacteria (98)					
**ENRO**	MIC_50_	< 0.03	0.13–1	0.06–1	[46]	81	0.003	1.19	0.038
	MIC_90_	0.13	0.25–2	0.06–1					
		Anaerobic bacteria (119)							
**SMX**	MIC_50_	2.4–53				239	0.0001	0.03	0.01
	MIC_90_	5.3 - > 128			[47]				
**TRIM**	MIC_50_	0.3–51				330	0.001	0.05	0.02
	MIC_90_	> 19.5–128							
		Neisseria Meningitidis strains							
**SDZ**	MIC	0.005–2			[48]	108	0.022	12.82	4.14

See Table 2 for PEC values; MIC_50_ (MIC_90_): MIC at which 50% (90%) of the strains were at or below

As shown in Table 2, the ratios of the maximum quantified concentrations to the PNEC values were lower than 1. Thus, the occurrence of the four studied antibiotics did not pose a risk to the aquatic environment in the studied systems. Moreover, quantified antibiotic concentrations in all collected water samples were approximately three or four orders of magnitude lower than the respective MIC values. However, the calculated PEC values for all four studied antibiotics were three orders of magnitude higher compared to the median quantified concentrations (Table 4). The PEC/PNEC ratios as well as the PEC/MIC ratios of SDZ and ENRO were higher than 1, stressing the importance of detailed investigations of the environmental fate of SDZ and ENRO for further risk assessment [44].

In summary, the studied antibiotics were found nearly ubiquitously in surface water, but as the background levels were low, they are unlikely to cause an overall risk to aquatic organisms, according to the current state of knowledge. However, detected background levels were influenced by dilution processes (rainfall, mixing of water sources with other water sources) and particularly by dissipation processes i.e. the monitoring could not cover maximum antibiotic loads immediately after application. Dissipation of organic pollutants like pesticides in tropical ecosystems may occur very rapidly (e.g., [49,50]). Since PEC/PNEC ratios of SDZ and ENRO were greater than 1, we conducted dissipation studies to better understand the fate of the antibiotics under tropical aquaculture conditions.

### Dissipation of SDZ, TRIM and ENRO in different semi-field experiment systems

#### Persistence of SDZ, TRIM and ENRO

**Fig 3:**
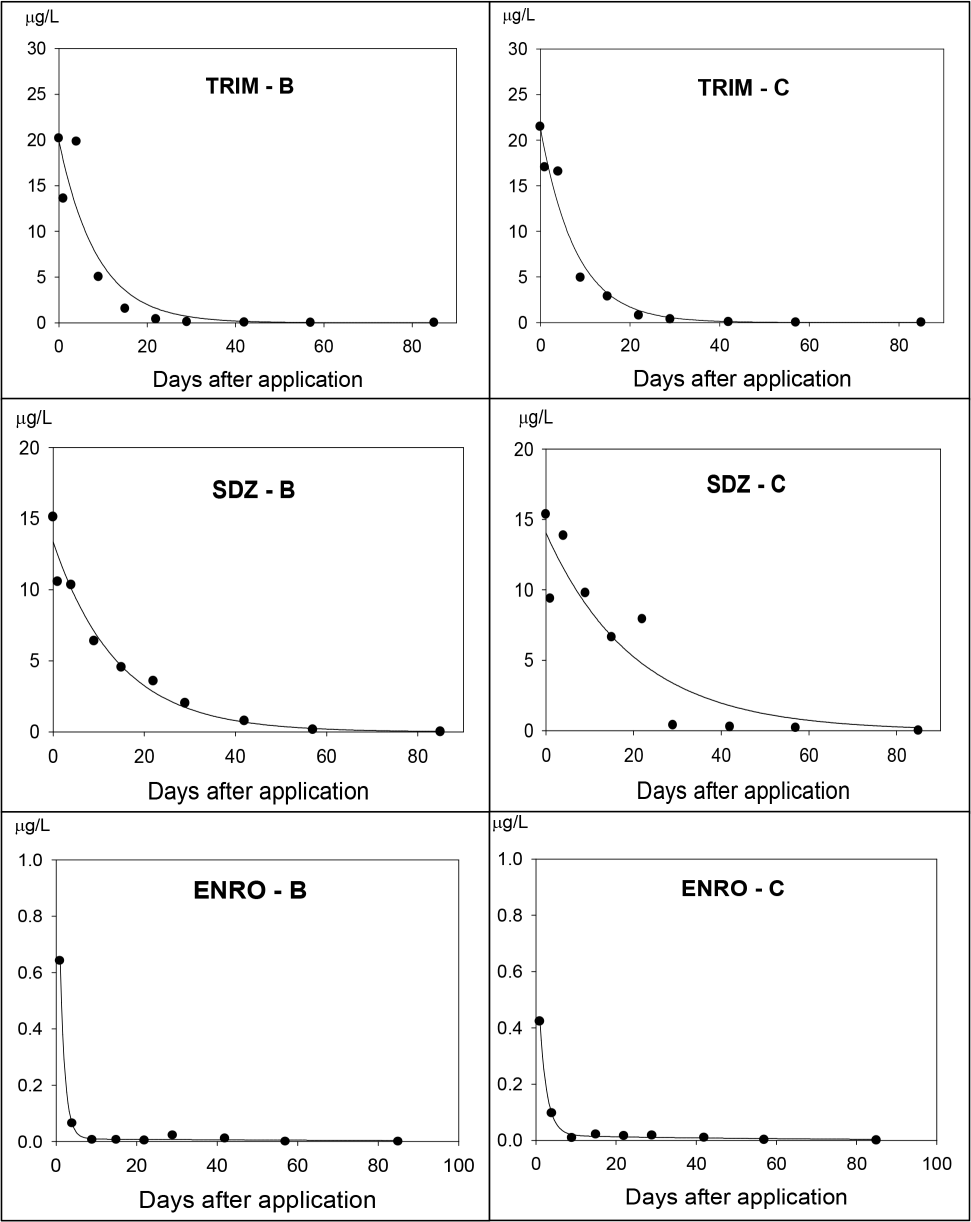
Dissipation curves of TRIM, SDZ and ENRO in water phase of the total water:sediment systems B (natural light regime) and C (light control). The concentration scales are different between compounds.

The dissipation of antibiotics was assessed using microcosm systems as described by Laabs et al. ([29], Fig 2). The dissipation profiles of the studied antibiotics followed simple first order kinetics and were fitted to exponential decay equations (see Fig 3 as an exemplary for dissipation curves of the target antibiotics). Exceptions were the dissipation of SDZ in the dark water system (D) and of ENRO in the total water:sediment system under a natural light regime due to a lack of clear temporal trend. For these systems, no dissipation half lives (DT_50_) were computed. Recovery rates immediately after application (day 0) of SDZ and TRIM in all microcosms varied from 60% to 79% and from 94% to 123%, respectively. In water microcosms, ENRO showed a better recovery rate of 60% (A) and 74% (D) compared with those in water:sediment microcosms where only 32% of the initial ENRO concentration was recovered. This likely reflected the strong sorption of this fluoroquinolone, probably accompanied by the formation of non-extractable residues, as recently reported for difloxacine in soils [14]. Yet, by definition these processes do not hamper the assessment of dissipation rates from the aqueous phase. The respective DT_50_ times ranged from 0.6 to 44.1 days (both for ENRO; Table 6), i.e, lying either in-between the monthly sampling intervals of the monitoring study or covering only a snapshot of it. In the later case, the monitored background concentrations may be significantly exceeded at selected sampling locations and immediately after application as assumed through the PEC calculation showed in Table 2 (see section Risk assessment). Hence, even if the overall contamination level was low according to commonly accepted risk criteria, this does not exclude the possibility of high local risks for short time intervals.

**Table 6:** Dissipation parameters of SDZ, TRIM and ENRO in different test systems.

Antibiotics	k	DT_50_	DT_90_	DT_99_	R^2^
	day^-1^	day	Day	day	
**Water phase**					
Natural light system (A)					
SDZ	0.1369	5.1	16.8	33.6	0.9121
TRIM	0.0238	29.1	96.7^*^	193.5^*^	0.9030
ENRO	1.1093	0.6	2.1	4.2	0.9337
Light control system (D)					
SDZ	-	-	-	-	-
TRIM	0.0228	30.4	101.0^*^	201.0^*^	0.6898
ENRO	0.5302	1.3	4.3	8.7	0.8407
Water:sediment—natural light system (B)					
SDZ	0.0798	9.8	32.5	65.0	0.9633
TRIM	0.1151	6.0	20.0	40.0	0.8602
ENRO	0.7649	0.9	3.0	6.0	0.9979
Water:sediment—light control system (C)					
SDZ	0.0493	14.1	46.7	93.4^*^	0.8364
TRIM	0.1265	5.5	18.2	36.4	0.9639
ENRO	0.5558	1.2	4.1	8.3	0.9945
**Total system**					
Water:sediment—natural light system (B)					
SDZ	0.029	23.9	79.4	158.8^*^	0.8566
TRIM	0.0588	11.8	39.2	78.3	0.7989
ENRO	-	-	-	-	-
Water:sediment—light control system (C)					
SDZ	0.0215	32.2	107.1^*^	214.2^*^	0.7426
TRIM	0.0862	8.0	26.7	53.4	0.9486
ENRO	0.0157	44.1	146.7^*^	293.3^*^	0.6951

^*^: outside of incubation period

- **Sulfadiazine (SDZ):** in the water phase, SDZ degraded faster in the transparent water system (A, DT_50_ = 5 days) than in the water:sediment systems (B, DT_50_ = 10 days; C, DT_50_ = 14 days). In this semi-field experiment, light was partly blocked by the suspended particles in system B and fully inhibited in system C. The results demonstrate the influence of sunlight on the decomposition rate of SDZ in water. Photodegradation of SDZ in water under laboratory incubation has been documented elsewhere (e.g., [51,52,53]). Dissipation rates of SDZ in total water:sediment systems were slower (B, DT_50_ = 24 days; C, DT_50_ = 32 days; Table 6). This finding confirmed the photolytic sensitivity of SDZ with a reduction of half-life in the total water:sediment system exposed to sunlight (B), but at the same time points to higher risks of SDZ for benthic microorganisms than for aquatic ones. Yang et al. [54] reported the influence of biodegradation on SDZ dissipation where the half-lives for SDZ in aerobic nonsterile soils ranged from 12 days to 18 days, while under anoxic conditions the half-lives ranged between 57 days and 237 days. The low K_oc_ and K_ow_ values of SDZ (Table 1) indicate weak sorption, high mobility and bioavailability in soil. However, the high organic matter content in the investigated sediment (S1 Table) also created favorable conditions for a stronger sorption of SDZ to the soil [5], which resulted in a longer dissipation time in the water:sediment microcosms (B and C) compared with water microcosm (A). As a result, in the total water:sediment systems, more than 13% of the spiked concentration of SDZ was still quantified on the last day of the incubation period (day 85).

- **Trimethoprim (TRIM):** Dissipation profiles again followed exponential decay curves (R^2^ varied from 0.69 to 0.96). Dissipation behavior of TRIM was different among systems compared with SDZ and ENRO. Photodegradation was unlikely to cause dissipation since there were no significant differences of TRIM concentrations between transparent and dark systems (Table 6). Also in the water phase, degradation rate of TRIM was fairly slow, with half-lives of around 30 days. Consequently, TRIM was the most persistent compound of the three studied antibiotics in the water system, and the 90% and 99% dissipation fractions of TRIM in water lay outside the incubation period. In contrast, a rapid dissipation of TRIM was observed in the water phase of the water:sediment systems (DT_50_ = 5.5 to 11.8 days; Table 6). As the K_oc_ and K_ow_ values of TRIM are low (Table 1), it can be assumed that biodegradation largely controlled the dissipation of TRIM. A study of Liu et al. [55] also showed a fast decomposition of TRIM under non-sterile conditions.

- **Enrofloxacin (ENRO):** of the three studied antibiotics, ENRO was the least persistent in water systems (A and D) (Table 6). In transparent water system A, ENRO rapidly decomposed immediately after application (DT_50_ < 1), and after 4 days of incubation, 99% of the initial spiked concentration had dissipated. Meanwhile in the dark water system D, 50% and 99% of ENRO dissipation fractions were recorded at day 1 and day 9, respectively. Fluoroquinolones have frequently been reported to show low biodegradation in the environment [56], but fast degradation with, e.g., pure fungal cultures (e.g., [16,17]). It is therefore a question of to what degree microorganisms affected the ENRO dissipation process.

The fast degradation of ENRO in system A could be subjected to the influence of light. Particularly under mildly alkaline conditions as prevailing in the test systems (pH value ca. 8, see S1 Fig), photodegradation of ENRO has been shown to be enhanced ([57]). However, photolysis sensitivity of ENRO in the water phase of water:sediment systems was unclear (DT_50_ recorded at about one day for both system B and C). As described above, it is likely that this did not reflect fast biodegradation but rather rapid sorption of the compound to the sediment phase. In the totally dark water:sediment system (C), ENRO performed as the most persistent antibiotic with a DT_50_ value of 44 days and the dissipation fractions of 90% and 99% exceeded the incubation time. The work of Boxall et al. [58] has also confirmed the high persistence of ENRO in soils and sediments (DT_50_ = 152 days). Once bound to sediment, however, the fluorquinolones may not necessarily be easily re-released to affect microbial communities [14]. Andrieu et al. [59] reported that ENRO residue concentrations after application probably did not cause severe toxic effects to exposed aquatic ecosystems.

#### Application of dissipation equations to natural water systems

At a given sampling point, based on the year-round fluctuation of the measured concentrations, any peak of concentration of an individual antibiotic recorded at a given sampling date would be assigned to the initial concentration (C_0_) of the dissipation equation. Accordingly, the antibiotic concentration measured in the next sampling month would be equal to the C_t_ of the respective equation. Predicted antibiotic concentrations based on the computed exponential decay equations were then compared with the measured concentrations in canal water. The findings show that for the site *Pangasius 2*, the dissipation equations of TRIM in water systems both with and without light control (A and D) predicted almost exactly (r = 0.96, Table 7) the measured concentrations of TRIM in canal water samples collected at points P2a and P2b. Only at sampling point P2c, there were differences between the predicted and measured antibiotic concentrations, which most likely reflected the influence of a more complex water network at that point. Also the low monitored concentrations of ENRO and SDZ at the points P2a and P2b of the site *Pangasius 2* were well predicted from the dissipation behavior in the water systems at the sampling dates (Table 7). Predicted antibiotic concentrations in canal water at the *Hatchery* sites did not correlate with measured concentrations. At these sites, various fingerling species (e.g carp, tench, tilapia, catfish) were cultivated, leading to mixing of the wastewater every 2 or 3 months. There was also no correlation at the *Pangasius 1* site, where the fish ponds were mainly discharged to the rice fields before reaching the canal water. Apparently, deviations from the simple prediction of concentrations using the dissipation functions can be seen as an indicator of mixed wastewater sources or indirect discharge in the canal.

**Table 7:** Comparison between measured concentrations (ng L-1) in canals (P2a and P2b) and predicted concentrations (ng L-1) based on dissipation equations.

TRIM	Natural light: C_t_ = C_0_ e^-0.0238t^				Light control: C_t_ = C_0_ e^-0.0228t^			
	P2a		P2b		P2a		P2b	
	measured	predicted	measured	predicted	measured	predicted	measured	predicted
C0	41	41	42	42	41	41	42	42
C24	32	23	29	24	32	24	29	24
C57	11	11	12	11	11	11	12	12
C0	37	37	25	25	37	37	25	25
C34	14	16	16	11	14	17	16	12
C66	1	8	1	5	1	8	1	6
Correlation R	0.96				0.96			
Estimated max. conc.		84		86		82		83
**ENRO**	Natural light: C_t_ = C_0_ e^-1.1093t^				Light control: C_t_ = C_0_ e^-0.5302t^			
	P2a		P2b		P2a		P2b	
	measured	predicted	measured	predicted	measured	predicted	measured	predicted
C0	59	59	43	43	59	59	43	0.043
C24	20	0	0	0	20	0	0	0
C57	0	0	0	0	0	0	0	0
C0			19	19			19	0.019
C34			0	0			0	0
C66			0	0			0	0
C88			0	0			0	0
**SDZ**	Natural light: C_t_ = C_0_ e^-0.1369t^							
	P2a		P2b					
	measured	predicted	measured	predicted				
C0			10	10				
C24			0	0				
C0	25	25		9				
C40	2	0		0				
C0			10	10				
C34			0	0				
C66			0	0				
C88			0	0				
Estimated max. conc.		1515		580				

To estimate the potential maximum antibiotic concentrations that could occur under given environmental conditions, the derived dissipation equations were used to back-calculate the initial antibiotic concentration at application (C_0_) from the highest concentration of the antibiotic detected at the site during the year (C_t_). A maximum of 30 days was assumed between the application of the antibiotic and the sample measurement (t = 30), to give a maximum initial application concentration.

Accordingly, the predicted maximum concentrations in canal water at the day of application of TRIM at the two *Pangasius* sampling points P2a and P2b was approximately 80 ng L^-1^; of SDZ was about 1515 ng L^-1^ at point P2a and 580 ng L^-1^ at point P2b (Table 7). For ENRO, its fast decomposition under natural water conditions (DT_50_ < 1) did not allow feasible maximum concentrations within 30 days of the sampling day to be provided. Nevertheless, the high detected concentrations of ENRO in canal water implied that a large amount of ENRO had been used and discharged from the surrounding fish farms close to the sampling date. Those estimated concentrations were lower than the PEC, PNEC and MIC values shown in Table 2 and Table 5. The difference between these extrapolated concentrations and the PECs (derived from worst-case assumption) could be caused by the following reasons: 1) only 30% of the water in the fish ponds was discharged to the environment at the *Pangasius* site; 2) a portion of the applied antibiotics was taken up by the fish; 3) a fish farm contained several fish ponds, and antibiotics would only be applied to a pond where sick fish might predominate, while the worst-case scenario considered a total amount of antibiotics applied for the entire farm.

In summary, these results confirm the low potential for antibiotic pollution in water sources in the Mekong Delta, which is unlikely to cause an overall risk to aquatic organisms. On the other hand, it is still an open discussion to what degree a long-term and frequent occurrence of lower antibiotic concentration levels may still facilitate the development of bacterial resistance (see [60], for a recent review).

## Conclusions

Among the main aquaculture producers in Asia, Vietnam had the highest reported number of different types of antibiotics in use [6]. This study also recorded widespread use of antibiotics in both hatcheries and mature pangasius farms. Local fish farmers’ knowledge of the properties or dosage of used antibiotics was not optimal; antibiotics have also been used for prophylaxes, without worker protection and without exact knowledge about the required dosage. The questionnaires revealed that applications mainly consisted of sulfonamides (like SMX, SDZ), jointly with TRIM, tetracyclines (not studied here), fluorquinolones (ENRO), and penicillins (also not studied here). This study also provides the first large scale background database on the presence of the four different currently used antibiotics SMX, SDZ, TRIM and ENRO in different drinking water sources in the Mekong Delta. The monitoring of surface waters, however, revealed that SMX, SDZ, TRIM and ENRO commonly occurred in the aquatic environment, but their concentrations were generally low in surface water. Concentrations of SMX and TRIM in the areas dominated by fish hatcheries were higher than in the area dominated by mature pangasius culture where wastewater was drained to surrounding rice fields both before being discharged to surrounding water systems (*Pangasius 1*). Nevertheless, since neither the measured nor the predicted concentrations reached critical PNEC or MIC values in any of the samples, it is suggested that, at least according to these toxicity standards, dilution by rain, river and canal water is sufficient to prevent high concentrations in surface waters.

After application, SDZ, TRIM and ENRO exhibited different dissipation patterns in water and water:sediment systems under tropical climate. The dissipation half-lives ranged from 1 to 44 days, depending on the availability of sunlight and sediment. TRIM was the most persistent antibiotic in water systems and not susceptible to photodegradation. Photolysis, however, affected the dissipation of ENRO and SDZ. While SDZ prevailed to some degree in water:sediment systems, thus being prone to long-distance transport, ENRO showed strong sorption to sediment after application, suggesting that benthic organisms may be more at risk than aquatic ones by these compounds. The good match between monitored and predicted antibiotic concentrations supports the assumption that the semi-field experimental design was suitable for assessing realistic dissipation rates. It helped to validate the models that could be considered as conceptual models for further risk assessment

Despite low concentrations of antibiotics in the canals as well as in the rivers, the large amount of water transported by the Mekong River (457 km^3^ per year, [61]) results in large overall loads of antibiotics (ENRO ca. 5800 tons, SDZ ca. 1800 tons, SMX 12300 tons, and TRIM ca. 6400 tons per year as a first rough estimate, based on the discharge and concentration detected in the Hau River (CTPS)). Hence, reducing overall loads of antibiotics is still an important policy requirement. Management measures to be considered should focus on 1) reducing fish stocking densities, 2) constructing sedimentation reservoirs for wastewater sedimentation and decomposition, and 3) education programs for local farmers, emphasizing the role of individuals in the context of sustainable aquaculture development, to raise awareness of proper antibiotic use and management, reverse effects from bacterial resistance, and legality of use.

## Supporting Information

S1 Table Basic characteristics of water and sediment of the fate study experiment. (PDF)

S2 Table Assessment factors to derive a PNEC_aquatic_ [45]. (PDF)

S1 Fig Differences of test system conditions. (PDF)

## References

[1] Food and agriculture organization of the United Nations (2012) The state of world fisheries and aquaculture 2012. Available: http://www.fao.org/docrep/016/i2727e/i2727e.pdf

[2] General Statistics Office (2012) Statistical yearbook of Vietnam 2012 Statistical Publishing House, Vietnam.

[3] Bosma RH, Hanh CTT, Potting J (2009) Environmental impact assessment of the pangasius sector in the Mekong Delta Wageningen University, Wageningen, The Netherlands.

[4] Lam PT, Tam BM, Thuy TTN, Gooley GJ, Ingram BA, Hao NV, et al (2009) Current status of farming practices of striped catfish, *Pangasianodon hypophthalmus* in the Mekong Delta, Vietnam. Aquaculture 296: 227–236.

[5] Thiele-Bruhn S (2003) Pharmaceutical antibiotic compounds in soils—a review. Journal of Plant Nutrition and Soil Science 166: 145–167.

[6] Rico A, Satapornvanit K, Haque MM, Min J, Phuong NT, Telfer TC, et al (2012) Use of chemicals and biological products in Asian aquaculture and their potential environmental risks: a critical review. Reviews in Aquaculture 4: 1–19.

[7] Tuan XL and Munekage Y (2004) Residues of selected antibiotics in water and mud from shrimp ponds in mangrove areas in Vietnam. Marine Pollution Bulletin 49: 922–929. 15556177 10.1016/j.marpolbul.2004.06.016

[8] Managaki S, Murata A, Takada H, Tuyen BC, Chiem NH (2007) Distribution of macrolides, sulfonamides and trimethoprim in tropical waters: ubiquitous occurrence of veterinary antibiotics in the Mekong Delta. Environmental Sciences & Technology 41: 8004–8010. 10.1021/es0709021 18186329

[9] General Statistics Office (2008) Results of the survey on household living standards 2008 Statistical publishing house, Vietnam.

[10] Reis N and Mollinga PP (2012) Water supply or ‘Beautiful latrines’? Microcredit for rural water supply and sanitation in the Mekong Delta, Vietnam. ASEAS–Austrian Journal of South-East Asian Studies 5(1): 10–29.

[11] Toan PV, Sebesvari Z, Bläsing M, Rosendahl I, Renaud FG (2013) Pesticide management and their residues in sediments and surface and drinking water in the Mekong Delta, Vietnam. Science of the Total Environment 452–453: 28–39. 10.1016/j.scitotenv.2013.02.026 23500396

[12] Wilbers GJ, Becker M, Nga LT, Sebesvari Z, Renaud FG (2014) Spatial and temporal variability of surface water pollution in the Mekong Delta, Vietnam. Science of the Total Environment 485–486: 653–665. 10.1016/j.scitotenv.2014.03.049 24747257

[13] Forster M, Laabs V, Lamshoft M, Groeneweg J, Zuhlke S, Spiteller M, et al (2009) Sequestration of manure-applied sulfadiazine residues in soils. Environmental Science & Technology 43: 1824–1830. 19368178 10.1021/es8026538

[14] Rosendahl I, Siemens J, Kindler R, Groeneweg J, Zimmermann J, Czerwinski S, et al (2012) Persistence of the fluoroquinolone antibiotic difloxacin in soil and lacking effects on N-turnover. Journal of Environmental Quality 41: 1275–1283. 10.2134/jeq2011.0459 22751072

[15] Jessick AM, Moorman TB, Coats JR (2013) Fate of erythromycin in sediment-containing surface water microcosms: how does aged erythromycin in sediment influence bioavailability? In: Cobb GB and Smith PN, editors. Evaluating veterinary pharmaceutical behavior in the environment. ACS Symposium Series 1126: 161–178.

[16] Wetzstein H-G, Schmeer N, Karl W (1997) Degradation of the fluoroquinolone enrofloxacin by the brown rot fungus gloeophyllum striatum: Identification of metabolites. Applied and Environmental Microbiology 63: 4272–4281. 9361414 10.1128/aem.63.11.4272-4281.1997 PMC168747

[17] Wetzstein H-G, Stadler M, Tichy H-V, Dalhoff A, Karl W (1999) Degradation of ciprofloxacin by basidiomycetes and identification of metabolites generated by the brown rot fungus gloeophyllum striatum. Applied and Environmental Microbiology 65 (4): 1556–1563. 10103250 10.1128/aem.65.4.1556-1563.1999 PMC91220

[18] Sukul P and Spiteller M (2007) Fluoroquinolone antibiotics in the environment. In: Ware GW, Nigg HN, Doerge DR, editors. Reviews of Environmental Contamination and Toxicology 191: 131–162. 17708074 10.1007/978-0-387-69163-3_5

[19] Bjorklund NV, Rabergh CMI, Bylund G (1991) Residues of oxolinic acid and oxytetracycline in fish and sediments from fish farms. Aquaculture 97: 85–96.

[20] Coyne R, Hiney M, O’Connorb B, Kerry J, Cazabona D, Smith P (1994) Concentration and persistence of oxytetracycline in sediments under a marine salmon farm. Aquaculture 123: 31–42.

[21] Nepejchalova L, Svobodova Z, Kolarova J, Frgalova K, Valova J, Nemethova D (2008) Oxytetracycline assay in pond sediment. Acta Veterinaria Brno 77: 461–466.

[22] Phong TK, Nhung DTT, Hiramatsu K, Watanabe H (2009) Prediction of the Fate of oxytetracycline and oxolinic acid in a fish pond using simulation model—A preliminary study. Journal of the Faculty of Agriculture, Kyushu University 54 (2): 513–521.

[23] Chau NDG, Sebesvari Z, Amelung W, Renaud F (2015) Pesticide pollution of multiple drinking water sources in the Mekong Delta, Vietnam: evidence from two provinces. Environmental Science and Pollution Research. 10.1007/s11356-014-4034-x 25572267

[24] Ciarlone AE, Fry BW, Ziemer DM (1990) Some observations on the adsorption of tetracyclines to glass and plastic labware. Microchemical Journal 42(2): 250–255.

[25] Dalkmann P, Broszat M, Siebe C, Willaschek E, Sakinc T, Huebner J (2012) Accumulation of pharmaceuticals, enterococcus, and resistance genes in soils irrigated with wastewater for zero to 100 years in central Mexico. PLoS ONE 7(9): e45397 10.1371/journal.pone.0045397 23049795 PMC3458031

[26] Drugbank (2013). Available: http://www.drugbank.ca/drugs/DB00359

[27] Li ZH, Randak T (2009) Residual pharmaceutically active compounds (PhACs) in aquatic environment—status, toxicity and kinetics: a review. Veterinarni Medicina 52(7): 295–314.

[28] University of Hertfordshire (2011) VSDB: Veterinary substances database. Last updated 2011. Available: http://sitem.herts.ac.uk/aeru/vsdb/1745.htm

[29] Laabs V, Wehrhan A, Pinto A, Dores E, Amelung W (2007) Pesticide fate in tropical wetlands of Brazil: an aquatic microcosm study under semi-field conditions. Chemosphere 67: 975–989. 17166548 10.1016/j.chemosphere.2006.10.067

[30] Gobel A, Thomsen A, McArdell CS, Alder AC, Giger W, Theiß N, et al (2005) Extraction and determination of sulfonamides, macrolides, and trimethoprim in sewage sludge. Journal of Chromatography A 1085 (2): 179–189. 16106697 10.1016/j.chroma.2005.05.051

[31] Golet EM, Strehler A, Alder AC, Giger W (2002) Determination of fluoroquinolone antibacterial agents in sewage sludge and sludge-treated soil using accelerated solvent extraction followed by solid-phase extraction. Analytical Chemistry 74: 5455–5462. 12433073 10.1021/ac025762m

[32] Dung TT, Ngoc NTN, Thinh NQ, Thy DTM, Tuan NA, Shinn A, et al. (2008) Common diseases of pangasius catfish farmed in Vietnam. Global Aquaculture Advocate: 77–78. Available: http://cenres.ctu.edu.vn/Publication/Tai%20lieu%20xuat%20ban%20quoc%20te%20nam%202008/07_tapchiquocte_2008_Nhu%20Ngoc.pdf

[33] Serrano PH (2005) Responsible use of antibiotics in aquaculture. FAO Fisheries Technical Paper 469. Rome, 97p.

[34] TT-BNN (2009) Circular to promulgate the list of drugs, chemicals, antibiotics banned for uses, limited to use Ministry of Agriculture and rural development, Vietnam Available: http://www.spsvietnam.gov.vn/Lists/VBPQ_EN/Attachments/480/15-2009-TT-BNN_Eng.pdf

[35] Phuong NT, Oanh DTH (2009) Striped catfish (*Pangasianodon hypophthalmus*) aquaculture in Viet Nam: an unprecedented development within a decade In: De Silva SS, Davy FB, editors. Success stories in Asian aquaculture. Springer, NACA and IDRC, Dordrecht, Bangkok and Ottawa, pp. 133–149.

[36] Carballo E, van Eer A, van Schie T, Hilbrands A (2008) Small-scale freshwater fish farming Agrodok 15. Agromisa Foundation and CTA, Wageningen, 84p.

[37] Riviere JE, Papich MG (2009) Sulfonamides and potentiated sulfonamides In: Riviere JE and Papich, editors. Veterinary Pharmacology and Therapeutics 9th edition, pp. 835–860.

[38] Lin AY, Yu TH, Lin CF (2008) Pharmaceutical contamination in residential, industrial, and agricultural waste streams: Risk to aqueous environments in Taiwan. Chemosphere 74: 131–141. 10.1016/j.chemosphere.2008.08.027 18829065

[39] Wang X, He X, Chen B, Xie C (2011) Rice field for the treatment of pond aquaculture effluents. African Journal of Biotechnology 10(34): 6456–6465.

[40] Trieu TTN, Lu M (2014) Estimates of nutrient discharge from striped catfish farming in the Mekong River, Vietnam, by using a 3D numerical model. Aquaculture International 22: 469–483.

[41] GSO (General Statistics Office) (2014) Statistical yearbook of Vietnam 2013 Statistical Publishing House, Vietnam.

[42] U. S. Environmental Protection Agency (2011) Finalization of guidance on incorporation of water treatment effects on pesticide removal and transformations in drinking water exposure assessments. Available: http://www.epa.gov/pesticides/science/efed/policy_guidance/team_authors/water_quality_tech_team/wqtt_dw_treatment_effects_removal_transformation.htm

[43] Luo Y, Guo W, Ngo HH, Nghiem LD, Hai FI, Zhang J, et al (2014) A review on the occurrence of micropollutants in the aquatic environment and their fate and removal during wastewater treatment. Science of the Total Environment 473–474: 619–641. 10.1016/j.scitotenv.2013.12.065 24394371

[44] Koschorreck J, Koch C, Ronnefahrt I (2002) Environmental risk assessment of veterinary medicinal products in the EU—a regulatory perspective. Toxicology Letters 131: 117–124. 11988364 10.1016/s0378-4274(02)00047-4

[45] European Commission (2003) Technical guidance document on risk assessment part II, pp:99–102. Available: https://echa.europa.eu/documents/10162/16960216/tgdpart2_2ed_en.pdf

[46] Salmon SA, Watts JL (2000) Minimum inhibitory concentration determinations for various antimicrobial agents against 1570 bacterial isolates from Turkey poults. Avian diseases 44: 85–98. 10737648

[47] Wust J, Wilkins TD (1978) Susceptibility of anaerobic bacteria to sulfamethoxazole/trimethoprim and routine susceptibility testing. Antimicrobial agents and chemotherapy 14(3): 384–390. 708016 10.1128/aac.14.3.384 PMC352469

[48] Wiggins GL, McLaughlin JV, Bickham ST, Jones WL, Balows A (1970) Susceptibility of Neisseria meningitidis strains from the civilian population to sulfadiazine, penicillin, and rifampin. Applied microbiology 20(6): 893–898. 4992654 10.1128/am.20.6.893-898.1970 PMC377079

[49] Ciglasch H, Busche J, Amelung W, Totrakool S, Kaupenjohann M (2006) Insecticide dissipation after repeated field application to a northern Thailand. Journal of Agricultural and Food Chemistry 54: 8551–8559. 17061833 10.1021/jf061521u

[50] Rosendahl I, Laabs V, Ahowe CA, James B, Amelung W (2009) Insecticide dissipation from soil and plant surfaces in tropical horticulture of southern Benin, West Africa. Journal of Environmental Monitoring 11:1157–1164. 10.1039/b903470f 19513446

[51] Boreen AL, Arnold WA, McNeill K (2004) Photochemical fate of sulfa drugs in the aquatic environment: sulfa drugs containing five-membered heterocyclic groups. Environmental Science and Technology 38: 3933–3940. 15298203 10.1021/es0353053

[52] Perisa M, Babic S, Skoric I, Fromel T, Knepper TP (2013) Photodegradation of sulfonamides and their N4-acetylated metabolites in water by simulated sunlight irradiation: kinetics and identification of photoproducts. Environmental Science and Pollution Research 20: 8934–8946. 10.1007/s11356-013-1836-1 23749364

[53] Lemanska-Malinowska N, Felis E, Surmacz-Gorska J (2013) Photochemical degradation of sulfadiazine. Archives of environmental protection 39(3): 79–91.

[54] Yang JF, Ying GG, Yang LH, Zhao JL, Liu F, Tao R, et al (2009) Degradation behavior of sulfadiazine in soils under different conditions. Journal of Environmental Science and Health Part B 44: 241–248. 10.1080/03601230902728245 19280477

[55] Liu F, Ying GG, Yang JF, Zhou LJ, Tao R (2010) Dissipation of sulfamethoxazole, trimethoprim and tylosinin a soil under aerobic and anoxic conditions. Environmental Chemistry 7: 370–376.

[56] Alexy R, Kumpel T, and Kummerer K (2004) Assessment of degradation of 18 antibiotics in the closed bottle test. Chemosphere 57: 505–512. 15350412 10.1016/j.chemosphere.2004.06.024

[57] Dolar D, Kosutic K, Perisa M, Babic S (2013) Photolysis of enrofloxacine and removal of its photodegradation products from water by reverse osmosis and nanofiltration membranes. Separation and Purification Technology 115: 1–8.

[58] Boxall ABA, Johnson P, Smith EJ, Sinclair CJ, Stutt E, Levy LS (2006) Uptake of veterinary medicines from soils into plants. Journal of Agricultural and Food Chemistry 54(6): 2288–2297. 16536609 10.1021/jf053041t

[59] Andrieu M, Rico A, Phu TM, Huong DTT, Phuong NT, van den Brink PJ (2015) Ecological risk assessment of the antibiotic enrofloxacin applied to Pangasius catfish farms in the Mekong Delta, Vietnam. Chemosphere 119: 407–414. 10.1016/j.chemosphere.2014.06.062 25063964

[60] Jechalke S, Heuer H, Siemens J, Amelung W, Smalla K (2014) Fate and effects of veterinary antibiotics in soil. Trends in Microbiology 22(9): 536–545. 10.1016/j.tim.2014.05.005 24950802

[61] Mekong River Commission (2009) The flow of the Mekong. MRC Management Information booklet series 2009. Available: http://www.mrcmekong.org/assets/Publications/report-management-develop/MRC-IM-No2-the-flow-of-the-mekong.pdf

