# Supplementary material for: Occurrence and Dissipation of the Antibiotics Sulfamethoxazole, Sulfadiazine, Trimethoprim, and Enrofloxacin in the Mekong Delta, Vietnam
Source: PLoS One. 2015 Jul 2;10(7):e0131855. doi: 10.1371/journal.pone.0131855 (PMC4489625; doi:10.1371/journal.pone.0131855)
Supplement: S1 Table — (PDF) [file pone.0131855.s001.pdf]

S1 Table. Basic characteristics of water and sediment of the fate study experiment. A: water: water system, no light control, B: water: sediment system, non light control, C: water: sediment system, light control D: water: water system, light control

**Water**	A	B	C	D
pH	8,56	7,17	7,05	7,82
EC	190	161	168	184
DO	10,15	5,63	2,73	6,43
Temp (^{0}C)	30,7	31,6	30,7	30,3
TN (mg L^{-1})	3,08	3,36	2,8	2,8
TP (mg L^{-1})	0,57	1,06	0,72	0,73
TOC (mg L^{-1})	2,09	2,78	2,77	2,39
**Sediment**		B	C	
pH_{water} (1:2.5)		5,07	4,97	
TC (CHC) (%C)		3,67	4,36	
TN (%)		0,13	0,15	
CEC (cmol kg^{-1})		14,2	13,7	
Density (g cm^{-3})		2,5	2,56	
