# Supplementary material for: Occurrence and Dissipation of the Antibiotics Sulfamethoxazole, Sulfadiazine, Trimethoprim, and Enrofloxacin in the Mekong Delta, Vietnam
Source: PLoS One. 2015 Jul 2;10(7):e0131855. doi: 10.1371/journal.pone.0131855 (PMC4489625; doi:10.1371/journal.pone.0131855)
Supplement: S2 Table — (PDF) [file pone.0131855.s002.pdf]

S2 Table. Assessment factors to derive a $PNEC_{aquatic}$ [45]

Available data	Assessment factor
At least one short-term $L(E)C_{50}$ from each of three trophic levels of the baseset (fish, Daphnia and algae)	1000
One long-term NOEC (either fish or Daphnia)	100
Two long-term NOECs from species representing two trophic levels (fish and/or Daphnia and/or algae)	50
Long-term NOECs from at least three species (normally fish, Daphnia and algae) representing three trophic levels	10
Species sensitivity distribution (SSD) method	5-1<br>(to be fully justified case by case)
Field data or model ecosystems	Reviewed on a case by case basis
