# Supplementary material for: Occurrence and Dissipation of the Antibiotics Sulfamethoxazole, Sulfadiazine, Trimethoprim, and Enrofloxacin in the Mekong Delta, Vietnam
Source: PLoS One. 2015 Jul 2;10(7):e0131855. doi: 10.1371/journal.pone.0131855 (PMC4489625; doi:10.1371/journal.pone.0131855)
Supplement: S1 Fig — (PDF) [file pone.0131855.s003.pdf]

S1 Fig. Differences of test system conditions

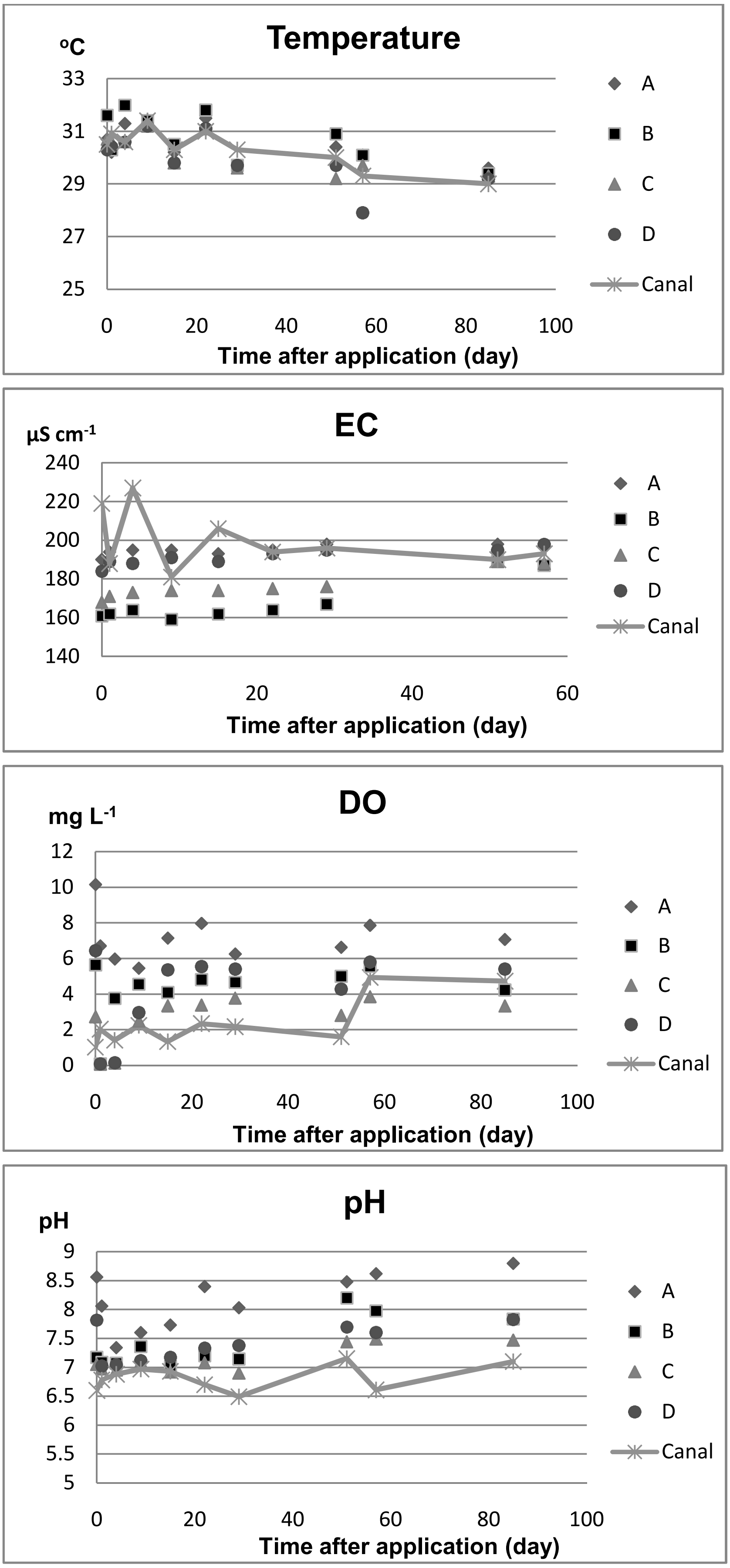

- *Temperature:* median water temperatures at -10 and -45 cm water depth of the canal recorded during the 10 sampling dates was 30.4 ± 0.7 ^{o}C, and varied from 30.1 ± 1 ^{o}C to 30.7 ± 0.9 ^{o}C for the 4 test systems. No depth gradient of water temperature was observed. There were also no significant difference of water temperatures between systems and between systems and canal. This implied the natural water temperature in the canal was well reflected in all the semi-field experiments.

- *Electrical conductivity (EC):*median EC values in water of system A (195 µS cm^{-1}) was significantly higher ($p < 0.05$) thanthat of systems B and C (water:sediment systems, median 164 µS cm^{-1}and 174 µS cm^{-}1, respectively). A similar pattern was recorded between water system D (191 µS cm^{-1}) and water:sediment system B, and between canal (194 µS cm^{-1}) and water:sediment system B and D. The low electrical conductivities in the water:sediment systems were mainly caused by the suspended particles released from the bottom sediment layer of the system during system movement forced by water waves or heavy rains, heavy winds. These particles hindered the electrical current, thus, conductivity level of water was decreased.

- *Dissolved oxygen (DO):*median DO value of system water A (6.9 ± 1.3 mg L^{-1}) was significant higher ($p < 0.05$) than that of system B (4.6 ± 1.6 mg L^{-1}), C (3.1 ± 1.4 mg L^{-1}) and the surrounding canal (2.1 ± 1.4 mg L^{-1}), but similar with system D(5.4 ± 2.3 mg L^{-1}).There were no significant differences between the other systems. After 9 incubation days, the DO values reached equilibrium and became stable until the end of the experiment (Figure 4). In the dark water:sediment system (C), the lack of sunlight restricted phytoplankton respiration which produced oxygen, and forced organisms in the system to consume dissolved oxygen and resulted in lowest oxygen levels. High DO values measured in water systems A and D were possibly linked to the water filtration process carried out while setting up the experiment. Filtration caused water disturbance which helped oxygen in the air to dissolve in water. It also increased the purity of canal water i.e. removed the amount of reductive materials or organisms which might consume the dissolved oxygen. Filtration on the one hand helped to partly removethe suspended solids in water (e.g. silt, small stones, small aquatic organisms) which was necessary for antibiotic analysis, but on the other hand changed the natural condition of canal water, in this case, the DO value. The low DO of canal water at the experimental site was mainly caused by the high silt content of the selected canal which is similar to almost river and canal systems in the Mekong Delta.

- *pH:* pH values recorded during the incubation period within the microcosms and the surrounding canal fluctuated from 7.3 to 8.8 in system A, 7.0 to 8.2 in system B, from 6.9 to 7.5in system C, from 7.0 to 7.8 in system D, and from 6.5 to 7.2 in canal. Median pH of canal water was also statistically significantly lower ($p<0.05$) compared with that of system A, B

and D. There was also a tendency for pH to rise in all test systems after day 29 of the experiment which could not be controlled in this semi-field study. A reasonable explanation would be linked to the water filtration process where a considerable amount of aerobic organisms or suspended organic materials in canal water were removed. In other words, normally, the respiration of living organisms and the decomposition of organic materials released carbon dioxide, lowering the pH of the media when combined with water. This process was limited in the water systems having pre-filtration that might cause a higher pH.

Temporal variation of water temperature, electrical conductivity (EC), dissolved oxygen (DO) and pH value of experiment microcosms during incubation time. *A: transparent water system, B: transparent water:sediment system, C: dark water:sediment system, D: dark water system.*
